# Comparison of deep vein thrombosis risks in acute respiratory distress syndrome caused by COVID-19 and bacterial pneumonia: a retrospective cohort study

**DOI:** 10.1186/s12959-022-00386-y

**Published:** 2022-05-10

**Authors:** Na Cui, Chunguo Jiang, Chenlu Yang, Liming Zhang, Xiaokai Feng

**Affiliations:** 1 Department of Respiratory and Critical Care Medicine, Beijing Institute of Respiratory Medicine and Beijing Chao-Yang Hospital, Capital Medical University, 8 Gongren Tiyuchang Nanlua, Chaoyang District, Beijing, 100020 China; 2 Department of Epidemiology and Biostatistics, Institute of Basic Medical Sciences Chinese Academy of Medical Sciences, School of Basic Medicine Peking Union Medical College, Beijing, China; 3 Department of Respiratory and Critical Care Medicine, Qinghai Provincial People’s Hospital, Qinghai University, 2 Gonghe Road, Chengdong District, Xining, 810000 Qinghai Province China

**Keywords:** Acute respiratory distress syndrome, Pneumonia, bacterial, COVID-19, Deep vein thrombosis

## Abstract

**Background:**

High incidence of deep vein thrombosis (DVT) has been observed in patients with acute respiratory distress syndrome (ARDS) caused by COVID-19 and those by bacterial pneumonia. However, the differences of incidence and risk factors of DVT in these two groups of ARDS had not been reported before.

**Study design and methods:**

We performed a retrospective cohort study to investigate the difference of DVT in incidence and risk factors between the two independent cohorts of ARDS and eventually enrolled 240 patients, 105 of whom with ARDS caused by COVID-19 and 135 caused by bacterial pneumonia. Lower extremity venous compression ultrasound scanning was performed whenever possible regardless of clinical symptoms in the lower limbs. Clinical characteristics, including demographic information, clinical history, vital signs, laboratory findings, treatments, complications, and outcomes, were analyzed for patients with and without DVT in these two cohorts.

**Results:**

The 28-days incidence of DVT was higher in patients with COVID-19 than in those with bacterial pneumonia (57.1% vs 41.5%, *P* = 0.016). Taking death as a competitive risk, the Fine-Gray test showed no significant difference in the 28-day cumulative incidence of DVT between these two groups (*P* = 0.220). Fine-Gray competing risk analysis also showed an association between increased CK (creatine kinase isoenzyme)-MB levels (*P* = 0.003), decreased PaO_2_ (partial pressure of arterial oxygen)/FiO_2_ (fraction of inspired oxygen) ratios (*P* = 0.081), increased D-dimer levels (*P* = 0.064) and increased incidence of DVT in COVID-19 cohort, and an association between invasive mechanical ventilation (IMV; *P* = 0.001) and higher incidence of DVT and an association between VTE prophylaxis (*P* = 0.007) and lower incidence of DVT in bacterial pneumonia cohort. The sensitivity and specificity of the corresponding receiver operating characteristic curve originating from the combination of CK-MB levels, PaO_2_/FiO_2_ ratios, and D-dimer levels ≥0.5 μg/mL were higher than that of the DVT Wells score (*P* = 0.020) and were not inferior to that of the Padua prediction score (*P* = 0.363) for assessing the risk of DVT in COVID-19 cohort.

**Conclusions:**

The incidence of DVT in patients with ARDS caused by COVID-19 is higher than those caused by bacterial pneumonia. Furthermore, the risk factors for DVT are completely different between these two ARDS cohorts. It is suggested that COVID-19 is probably an additional risk factor for DVT in ARDS patients.

**Supplementary Information:**

The online version contains supplementary material available at 10.1186/s12959-022-00386-y.

## Introduction

Deep vein thrombosis (DVT) and pulmonary embolism (PE), collectively referred to as venous thromboembolism (VTE), constitute a major global burden of disease [1]. Recent data have suggested that Coronavirus disease 2019 (COVID-19), like some other viruses, may impact the hematopoietic and hemostatic systems, resulting in thrombotic and bleeding complications [2–5], especially in critically ill COVID-19 patients. Studies have found that, even if VTE prophylaxis is given, the incidence rate of VTE is still high in these patients [6–8]. Also, our previous study confirmed that infection with severe acute respiratory syndrome coronavirus 2 (SARS-CoV-2) might increase VTE risk, and that the incidence of DVT increases rapidly with disease progression [9]. In addition, we found a high incidence of DVT in patients with acute respiratory distress syndrome (ARDS) caused by bacterial pneumonia [10]. Moreover, studies also found that the occurrence of DVT is associated with a poor prognosis in patients with ARDS [9, 10]. Thus, identifying the risk factors for DVT in this population is of crucial importance.

However, the DVT risks in ARDS have not been compared between patients with COVID-19 and those with bacterial pneumonia. The aim of this study therefore was to compare the incidence and risks of DVT between patients with ARDS caused by COVID-19 and those caused by bacterial pneumonia, and to further test that the COVID-19 is an additional risk factor [11].

## Methods

### Study design and population

This retrospective cohort study included COVID-19 subjects (from our previous cohort [9]) who were confirmed by laboratory tests (rhinopharyngeal specimen reverse transcription polymerase chain reaction test positive for SARS CoV-2). All subjects were hospitalized in the West Branch of Union Hospital (affiliated with Tongji Medical College, Huazhong University of Science and Technology), Wuhan, China, between January 29, 2020, and February 29, 2020. At that time, the hospital was the major designated referral and treatment hospital for critically ill adult COVID-19 patients (≥18 years old) that was following the World Health Organization’s interim guidance [12]. Considering bacterial pneumonia ARDS cases (≥18 years old), some were from our previous cohort [10], while some were from a single-center retrospective cohort study premiered at Beijing Chao-Yang Hospital, Beijing, China. All of these cases were confirmed by laboratory test results, and corresponding patients were hospitalized between January 1, 2015, and June 30, 2021. All of the patients met the criteria of the Berlin definition for diagnosis of ARDS [13].

The exclusion criteria were: active malignant tumor, cerebral stroke, acute myocardial infarction, serious trauma (injury severity score > 16), major operation lasting longer than 45 minutes, fracture of the lower limb, and joint replacement of hip or knee within 1 month before admission. Patients with a survival time less than 3 days and patients without lower extremity venous compression ultrasound data were also excluded.

**Fig. 1:**
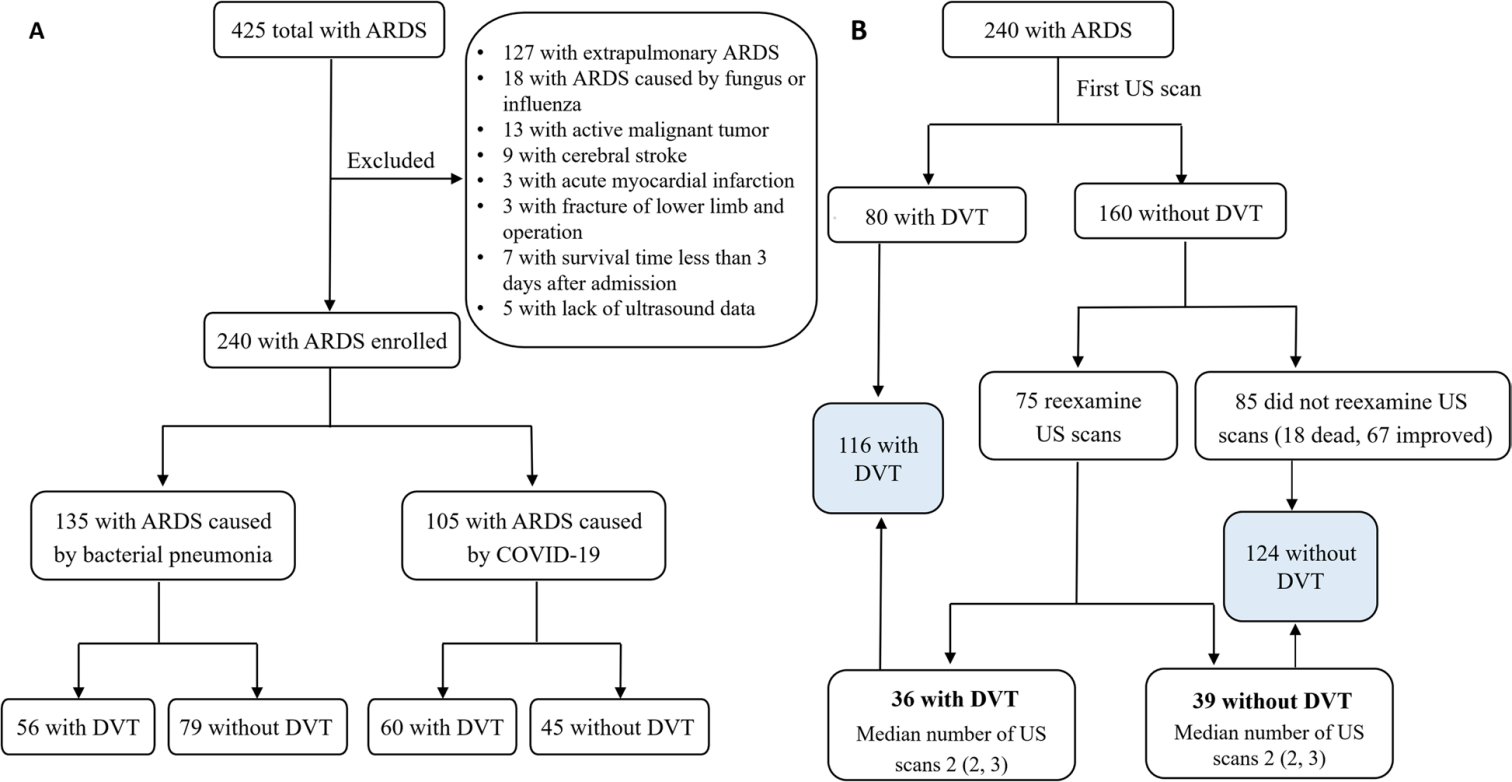
(**A, B**) Study flow chart. **A** flow chart for including patients; **B** flow chart for screening for DVT. The interval from the diagnosis of ARDS to the occurrence of DVT in the DVT group was 7 (4, 12) days, and the interval from the diagnosis of ARDS to the last ultrasound examination in the non-DVT group was 8 (3, 14) days. There were no differences between the two groups (*P* = 0.725). Abbreviations: ARDS, acute respiratory distress syndrome; COVID-19, coronavirus disease 2019; DVT, deep vein thrombosis; US, ultrasound

The first ultrasound examination was performed within 1–3 days after the diagnosis of ARDS. After intensive treatment, if the patient’s condition was unstable (e.g., due to unexplained hypoxemia or cardiac insufficiency), ultrasound was performed again. If there was more than one ultrasound scan for a single patient, all the results were recorded. Patients were divided into a DVT and a non-DVT group according to the results of the venous compression ultrasound of the lower extremities. The study flow chart is shown in Fig. 1 (A, B).

The study was approved by the Union Hospital, affiliated with Tongji Medical College, Huazhong University of Science and Technology (2020–0197), and the ethics committees of the Beijing Chao-Yang Hospital (2020-ke-429), and was conducted in accordance with the 1964 Helsinki Declaration and its later amendments or comparable ethical standards.

### Clinical data

Medical records, including demographic information, clinical history, vital signs, laboratory findings, treatments, complications, and outcomes of the patients during hospitalization, were collected and analyzed for all patients. We also analyzed the survival rates of all patients within 28 days after a diagnosis of ARDS. For patients discharged within 28 days, we followed up by phone concerning their survival status after discharge.

### Ultrasound assessment

Bedside ultrasound examinations were performed using a portable color ultrasound scanner (CX50, Philips Medical Systems, the Netherlands, equipped with an L12–3/S5–1 probe or EPIQ 7C, Philips Medical Systems, Andover, MA, equipped with an L12–5/S5–1 probe or a Mindray portable Ultrasound M9, GD, China, equipped with an L10–3 probe). The lower extremity venous compression ultrasound was obtained from the institution’s Picture Archiving and Communication System. The levels of DVT included the bilateral common femoral, deep and superficial femoral veins, the popliteal veins, and the anterior tibial, posterior tibial, peroneal, and calf muscle veins.

### Definitions

ARDS was defined according to the Berlin definition [13]. COVID-19 was diagnosed according to the Chinese Management Guideline for COVID-19 (version 6.0) [14]. Bacterial pneumonia, including community-acquired pneumonia and hospital-acquired pneumonia, was diagnosed according to the Clinical Practice Guidelines of the Infectious Diseases Society of America and the America Thoracic Society [15, 16]. A distal thrombosis was defined as thrombosis in the veins of the calf muscle or in at least 1 branch of the 3 pairs of deep calf veins (anterior tibial vein, posterior tibial vein, or peroneal vein); a proximal thrombosis was defined as thrombosis in the popliteal vein or above. The Padua prediction score was defined according to the Barbar model [17]. The Wells score for DVT was defined according to the Di Nisio model [1]. We applied the Acute Physiology and Chronic Health Evaluation (APACHE) II score and the Sequential Organ Failure Assessment (SOFA) score to assess the severity of the disease [18, 19].

### Statistical analyses

Categorical variables were described as number and percentage (%) and continuous variables, like mean, standard deviation, median, and interquartile range. The Shapiro-Wilk test was used to evaluate if a continuous variable follows a normal distribution. Differences between the DVT and the non-DVT groups were assessed by a two-sample *t*-test for normally distributed continuous variables, while the Mann-Whitney *U* test was used for non-normally distributed continuous variables; the χ^2^ or Fisher exact test was used for categorical variables. The variables with *P* <  0.2 based on univariate analysis were used as candidate variables of the Fine-Gray model. At the same time, combined with the prior research knowledge of pneumonia caused by bacteria and viruses, the model was further determined. Finally, the model was further perfected based on the Bayesian information criterion (BIC). Death was used as the competitive risk, and 28-day cumulative incidence curves (points estimates with 95% confidence interval [CI]) for COVID-19 and bacteria ARDS patients were plotted. The ine-Gray competitive risk model was used to explore the risk factors of DVT under COVID-19 and bacterial pneumonia subgroups. The adjusted hazards ratio (HR) with 95% CI was reported. To further evaluate the observed differences in risk factors for DVT between COVID-19 and bacterial pneumonia, we utilized interaction terms between ARDS type and each risk factor. A receiver operating characteristic (ROC) analysis was performed to calculate the sensitivity and specificity of risk factors for screening for DVT.

The comparison methods of diagnostic accuracy for screening for DVT in the ARDS cohort caused by COVID-19 were: patients were split by generating random numbers to produce a training data set (n*0.7) and a validation data set (n*0.3). The area under receiver operating curves (ROC-AUCs) for different risk factors were compared using the method of DeLong et al [20]. In order to enhance the practicability of the prediction model, we drew a nomogram based on the predictors selected from the COVID-19 ARDS population. Calibration was evaluated with calibration plots, which used the bootstrap method of 1000 resampling to show the relationship between the observed frequency and the prediction probability through a graph. In a well-calibrated model, the prediction should fall on a 45-degree diagonal.

All statistical analyses were performed using the Statistical Analysis System, version 9.4 (SAS Institute, Cary, NC, USA). All tests were two-tailed; *P* <  0.05 was considered statistically significant.

## Results

A total of 240 patients with ARDS were enrolled in this study; 105 patients were considered to belong to the COVID-19 ARDS cohort and 135 patients to the bacterial pneumonia ARDS cohort. The study flow chart is shown in Fig. 1 (A, B). We followed up the survival rates of all patients within 28 days after a diagnosis of ARDS. No patients were lost to follow-up.

### Ultrasound scan for screening for DVT

Lower extremity venous compression ultrasound scanning was performed for 240 patients regardless of clinical symptoms of the lower limbs (Fig. 1B). The median number of ultrasound scans was 1 (range, 1–5). Eighty (80/240) developed DVT was found and the other 160 was a negative result at the first ultrasound scan. Subsequently, 75 patients underwent more than one ultrasound scan; among those, 36 developed DVT and 39 had no DVT with 2 (range, 2–5) ultrasound examinations. The interval from the diagnosis of ARDS to the occurrence of DVT for the 36 patients who developed DVT was 8 (3, 14) days; the interval from the diagnosis of ARDS to the last ultrasound examination for the 39 cases with no DVT was 10 (5, 16) days. There was no difference between the two groups (*P* = 0.344).

Finally, of the 240 patients, 116 (48.3%) developed DVT, including 22 with proximal DVT and 94 with distal DVT, 77 of whom had muscular calf vein thrombosis only. The incidence of asymptomatic DVT was 94 (39.2%), including 15 (6.3%) proximal DVT and 79 (32.9%) distal DVT, of whom muscular calf vein thrombosis accounted for 67 (27.9%). For all the 240 patients, the interval from the diagnosis of ARDS to the occurrence of DVT in the DVT group was 7 (4, 12) days, and the interval from the diagnosis of ARDS to the last ultrasound examination in the non-DVT group was 8 (3, 14) days. There was no difference between the two groups (*P* = 0.725). In addition, six patients were clinically suspected of having PE; 4 were further confirmed by computed tomography pulmonary angiography (CTPA) examination (Table 1 and Supplementary-Table 1).

**Table 1:** Demographic and clinical characteristics of patients with ARDS caused by COVID-19 and bacterial pneumonia

Characteristic	Total (***N*** = 240)	Bacterial pneumonia (***N*** = 135)	COVID-19 (***N*** = 105)	***P*** value
Age, y	64.3 ± 14.2	64.8 ± 15.1	63.6 ± 13.1	0.522
Male	161 (67.1)	101 (74.8)	60 (57.1)	0.004
BMI, kg/m^2^	23.6 ± 3.4	23.7 ± 3.9	23.6 ± 2.7	0.977
Bed time ≥ 3 days	189 (78.8)	117 (86.7)	72 (68.6)	0.001
Hospital stays, d	23 (13, 38)	18 (11, 29)	31 (18, 41)	< 0.001
ARDS to DVT or last US scan, d	7 (3, 13)	5 (2, 12)	10 (6, 14)	< 0.001
Median number of US scans	1 (1, 2)	1 (1, 2)	1 (1, 1)	< 0.001
DVT Wells score	1 (1, 2)	1 (1, 1)	1 (0, 2)	0.004
Padua prediction score	5 (5, 6)	5 (5, 6)	5 (4, 6)	0.171
APACHE II score	16 (12, 23)	22 (17, 27)	11 (11, 13)	< 0.001
SOFA score	5 (4, 10)	6 (4, 10)	4 (3, 12)	0.012
Underlying disease				
Smoke	84 (35.0)	76 (56.3)	8 (7.6)	< 0.001
Chronic respiratory disease	32 (13.3)	25 (18.5)	7 (6.7)	0.007
Hypertension	103 (42.9)	60 (44.4)	43 (41.0)	0.588
Coronary heart disease	39 (16.3)	24 (17.8)	15 (14.3)	0.467
Diabetes	54 (22.5)	33 (24.4)	21 (20.0)	0.413
Cerebral vascular disease	31 (12.9)	27 (20.0)	4 (3.8)	< 0.001
Chronic liver disease	5 (2.1)	2 (1.5)	3 (2.9)	0.656
Chronic kidney disease	16 (6.7)	13 (9.6)	3 (2.9)	0.037
Symptoms of onset				
Fever	222 (92.5)	128 (94.8)	94 (89.5)	0.123
Cough	188 (78.3)	114 (84.4)	74 (70.5)	0.009
Dyspnea	198 (82.5)	130 (96.3)	68 (64.8)	< 0.001
DVT symptoms	46 (19.2)	27 (20.0)	19 (18.1)	0.710
Edema of lower extremities	42 (17.5)	27 (20.0)	15 (14.3)	0.248
Leg pain	6 (2.5)	2 (1.5)	4 (3.8)	0.408
Arterial blood gas analysis				
PaO_2_/FiO_2_, mm Hg	135 (81, 195)	137 (80, 188)	130 (81, 197)	0.858
Hematologic and infection-related indices				
White blood cell count, × 10^9^/L	10.9 (7.23, 16.0)	14.4 (10.1, 19.0)	8.1 (5.7, 10.9)	< 0.001
Neutrophil count, ×10^9^/L	9.7 (5.9, 14.2)	12.8 (9.0, 17.3)	6.5 (4.2, 9.6)	< 0.001
Lymphocyte count, × 10^9^/L	0.8 (0.5, 1.2)	0.8 (0.5, 1.2)	0.8 (0.5, 1.1)	0.528
Neutrophil-to-lymphocyte ratio	12.5 (7.1, 21.0)	15.1 (8.7, 23.7)	8.9 (4.7, 15.1)	< 0.001
Platelet count, ×10^9^/L	190 (133, 263)	185 (115, 261)	190 (144, 270)	0.205
Hemoglobin, g/L	116 (99, 130)	112 (87, 130)	118 (108, 132)	0.021
C-reactive protein, mg/L	99.5 (51.8, 120.0)	120.0 (82.0,120.0)	58.0 (20.7, 99.5)	< 0.001
Serum procalcitonin, ng/L	0.8 (0.1, 4.2)	3.2 (1.2, 11.3)	0.1 (0.1, 0.4)	< 0.001
Biochemical test				
Total protein, g/L	57.6 (50.9, 63.2)	53.0 (47.0, 59.0)	60.8 (57.6, 65.3)	< 0.001
Albumin, g/L	26.3 (23.6, 29.9)	25.3 (23.0, 29.6)	27.3 (24.2, 30.2)	0.007
Aspartate aminotransferase, U/L	35.0 (25.5, 58.0)	40.8 (26.0, 71.7)	33.0 (24.0, 44.0)	0.008
Alanine aminotransferase, U/L	33.2 (19.6, 60.2)	29.7 (17.9, 58.8)	35.0 (26.0, 62.0)	0.101
Total bilirubin, μmol/L	14.2 (10.1, 20.3)	15.2 (10.7, 22.8)	13.6 (9.2, 17.0)	0.006
Direct bilirubin, μmol/L	4.8 (3.1, 7.3)	5.2 (3.3, 8.4)	4.5 (3.0, 6.2)	0.038
Lactate dehydrogenase, U/L	354.0 (234.3, 546.0)	350.0 (239.0, 624.8)	354.0 (224.0, 511.0)	0.360
Blood urea nitrogen, mmol/L	7.54 (4.80, 13.78)	10.21 (5.39, 17.42)	6.50 (4.21, 9.21)	< 0.001
Serum creatinine, μmol/L	73.5 (56.7, 125.8)	89.1 (62.4, 193.0)	64.3 (53.7, 75.5)	< 0.001
eGFR, mL/min/1.73m^2^	88.3 (44.2, 104.2)	71.6 (29.2, 102.0)	95.6 (80.8, 106.9)	< 0.001
CK-MB, U/L	16.2 (10.8, 29.7)	16.2 (11.0, 26.9)	16.2 (10.0, 31.0)	0.812
Coagulation function				
D-dimer, μg/mL	1.8 (0.7, 4.6)	1.5 (0.6, 2.6)	2.8 (1.1, 8.0)	< 0.001
Prothrombin time, s	13.6 (12.60, 15.1)	13.5 (12.3, 15.2)	13.6 (12.7, 14.9)	0.193
Activated partial thromboplastin time, s	33.7 (29.7, 38.1)	32.1 (28.7, 35.7)	34.8 (32.5, 39.2)	< 0.001
DVT	116 (48.3)	56 (41.5)	60 (57.1)	0.016
Proximal DVT	22 (9.2)	6 (4.4)	16 (15.2)	0.004
Distal DVT	94 (39.2)	50 (37.0)	44 (41.9)	0.443
Muscular calf vein thrombosis only	77 (32.1)	38 (28.1)	39 (37.1)	0.139
Treatments				
Glucocorticoid therapy	90 (37.5)	40 (29.6)	50 (47.6)	0.004
Immunoglobulin therapy	56 (23.3)	3 (2.2)	53 (50.5)	< 0.001
CVC	82 (34.2)	45 (33.3)	37 (35.2)	0.758
CRRT	22 (9.2)	12 (8.9)	10 (9.5)	0.866
IMV	103 (42.9)	79 (58.5)	24 (22.9)	< 0.001
Sedative therapy	86 (35.8)	62 (45.9)	24 (22.9)	< 0.001
Vasoactive drugs	64 (26.7)	27 (20.0)	37 (35.2)	0.008
VTE prophylaxis	137 (57.1)	64 (47.4)	73 (69.5)	0.001
LMWH	117 (48.8)	55 (40.7)	62 (59.0)	0.005
LMWH + physical	80 (33.3)	40 (29.6)	40 (38.1)	0.168
Physical prophylaxis only	19 (7.9)	8 (5.9)	11 (10.5)	0.195
28-day mortality	73 (30.4)	46 (34.1)	27 (25.7)	0.163

Data are presented as mean ± SD, median (IQR), or n (%). *P* values comparing DVT and non-DVT groups were from a two-sample *t-*test, Mann-Whitney *U* test, χ^2^ test, or Fisher exact test. *P* < 0.05 was considered statistically significant

Abbreviations: *APACHE* Acute Physiology and Chronic Health Evaluation, *ARDS* acute respiratory distress syndrome, *BMI* body mass index, *CK* creatine kinase isoenzyme, *COVID-19* coronavirus disease 2019, *CRRT* continuous renal replacement therapy, *CVC* central venous catheterization, *DVT* deep venous thrombosis, *eGFR* estimated glomerular filtration rate, *FiO*_*2*_ a fraction of inspired oxygen, *IMV* invasive mechanical ventilation, *IQR* interquartile range, *LMWH* low molecular weight heparin, *PaO*_*2*_ partial pressure of arterial oxygen, *PE* pulmonary embolism, *SD* standard deviation, *SOFA* Sequential Organ Failure Assessment, *US* ultrasound, *VTE* venous thromboembolism

### Demographic and clinical characteristics of patients in COVID-19 and bacterial pneumonia ARDS cohorts

Of the 240 patients with ARDS, 105 were infected with COVID-19 (age [63.6 ± 13.1] years, male 60 [57.1%]) and 135 with bacterial pneumonia (age [64.8 ± 15.1] years, male 101 [74.8%]). Compared with patients with bacterial pneumonia, the rate of underlying diseases (smoke, chronic respiratory disease, cerebral vascular disease, and chronic kidney disease), APACHE II scores, and SOFA scores (all *P* <  0.05) were all lower in patients with COVID-19. There was no difference in PaO_2_/FiO_2_ ratios between two groups (*P* = 0.858). More patients with COVID-19 received therapy of glucocorticoids (47.6% [50/105] vs 29.6% [40/135], *P* = 0.004), immunoglobulin (50.5% [53/105] vs 2.2% [3/135], *P* <  0.001), vasoactive drugs (35.2% [37/105] vs 20.0% [27/135], *P* = 0.008) and VTE prophylaxis (69.5% [73/105] vs 47.4% [64/135], *P* = 0.001). Of the 105 patients with COVID-19, 73 (69.5%) were given VTE prophylaxis; among whom, 62 (59.0%) received low molecular weight heparin, 11 (10.5%) only received physical prevention, no patient received anticoagulant instead of LMWH, and 40 (38.1%) received combined treatment with LMWH and physical prevention. Of the 135 patients with bacterial pneumonia, 64 (47.4%) were given VTE prophylaxis; among those, 55 (40.7%) received LMWH, 8 (5.9%) only received physical prevention, 1 (0.7%) received rivaroxaban as anticoagulation drug, and 40 (29.6%) received combined treatment with LMWH and physical prevention. There was significantly higher incidence of DVT (57.1% vs 41.5%; *P* = 0.016) and proximal DVT (15.2% vs 4.4%; *P* = 0.004) in patients with COVID-19 than in those with bacterial pneumonia (Table 1).

**Fig. 2:**
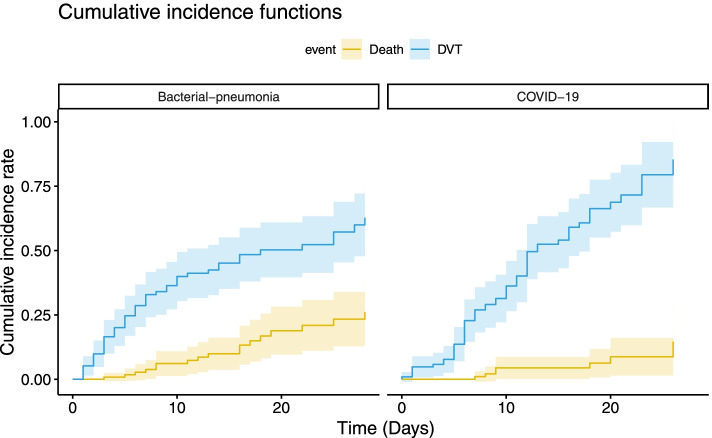
The 28-day cumulative incidence curves of DVT and 28-day cumulative death curves in COVID-19 and bacterial pneumonia ARDS cohorts. Based on univariate test results and prior knowledge, death was used as the competitive risk. The Fine-Gray test showed no significant difference in the 28-day cumulative incidence of DVT between the COVID-19 ARDS and bacterial pneumonia ARDS group (*P* = 0.220). Abbreviations: ARDS, acute respiratory distress syndrome; COVID-19, coronavirus disease 2019; DVT, deep vein thrombosis

Next, based on univariate test results and prior knowledge, death was used as a competitive risk. Fine-Gray model showed that the 28-day cumulative incidence rate (95% CI) of DVT in patients with ARDS caused by COVID-19 and by bacterial pneumonia was 85.3% (66.6, 92.3%) and 62.7% (48.1, 72.0%) respectively. There was no significant difference between the two groups (*P* = 0.220) (Fig. 2).

### Demographic and clinical characteristics of DVT vs non-DVT patients in overall ARDS cohort

Among 240 patients with ARDS (Supplementary Table 1), patients with DVT were older, and had longer bedridden time, higher Padua prediction scores, higher SOFA scores, lower serum creatinine levels, higher lactate dehydrogenase (LDH) levels, higher D-dimer levels, longer prothrombin time (PT), and lower PaO_2_/FiO_2_ ratios compared to patients without DVT (all *P* <  0.05). Also, more patients with DVT received therapy of glucocorticoids, immunoglobulin, vasoactive drugs, sedatives and invasive mechanical ventilation (IMV) (all *P* <  0.05). Moreover, patients with DVT had a significantly higher 28-day mortality (42.2% [49/116] vs 19.4% [24/124], respectively; *P* <  0.001) (Supplementary Table 1).

### Demographic and clinical characteristics of DVT vs non-DVT patients in COVID-19 and bacterial pneumonia ARDS cohorts

Among 105 COVID-19 patients with ARDS, patients with DVT were older, had longer bedridden time, higher Well scores, higher SOFA scores, higher WBC counts, higher neutrophil counts, higher neutrophil-to-lymphocyte ratios, higher serum procalcitonin (PCT) levels, higher AST levels, higher alanine aminotransferase (ALT) levels, higher TBIL levels, higher DBIL levels, higher LDH levels, higher BUN levels, higher creatine kinase isoenzyme (CK)-MB levels, higher D-dimer levels, longer PT, and lower PaO_2_/FiO_2_ ratios compared to patients without DVT (all *P* <  0.05). Also, more patients with DVT received central venous catheterization (CVC), therapy of vasoactive drugs, sedatives, and IMV (all *P* <  0.05). There was no significant difference in serum creatinine levels and proportion of VTE prophylaxis between DVT and non-DVT groups (all *P* > 0.05). Patients with DVT had a significantly higher 28-day mortality (33.3% [20/60] vs 15.6% [7/45], respectively; *P* = 0.039) (Supplementary Table 2).

Among 135 patients with ARDS caused by bacterial pneumonia, patients with DVT had higher serum PCT levels and lower PaO_2_/FiO_2_ ratios, and more patients with DVT had the underlying chronic respiratory disease and received sedative therapy and IMV compared with patients without DVT (all *P* <  0.05). Also, there were significantly higher serum creatinine levels in patients without DVT (*P* = 0.015). Patients with DVT also had a significantly higher 28-day mortality (51.8% [29/56] vs 21.5% [17/79], respectively; *P* <  0.001) (Supplementary Table 3).

### Independent risk factors associated with DVT in patients with ARDS caused by COVID-19 and bacterial pneumonia

**Fig. 3:**
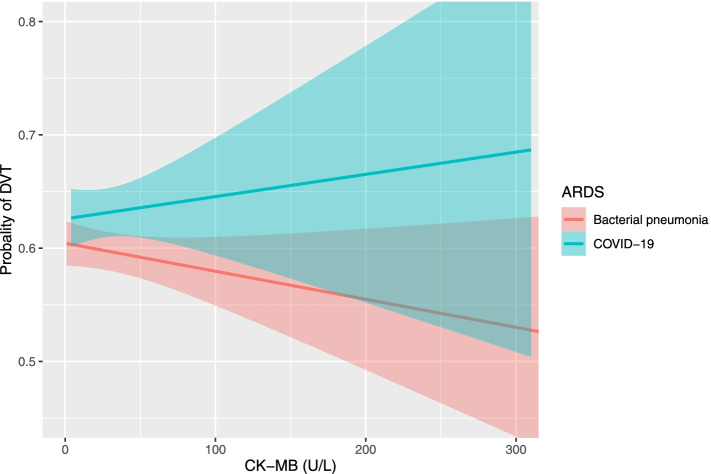
Probability of DVT increased with CK-MB levels only in the COVID-19 ARDS group. The occurrence of DVT in the COVID-19 ARDS group (green line) increased with the rising of CK-MB levels, whereas there was no association between DVT and CK-MB levels in the bacterial pneumonia ARDS group (red line; test for interaction, *P* = 0.016). Data are adjusted for age, serum creatinine levels, serum PCT levels, D-dimer levels, PaO_2_/FiO_2_ ratios, and IMV. Abbreviations: ARDS, acute respiratory distress syndrome; CK, creatine kinase isoenzyme; COVID-19, coronavirus disease 2019; DVT, deep vein thrombosis; FiO_2_, a fraction of inspired oxygen; IMV, invasive mechanical ventilation; PaO_2_, partial pressure of arterial oxygen; PCT, procalcitonin

Based on univariate test results and prior knowledge, death was used as a competitive risk. The Fine-Gray competitive risk model was used to explore the risk factors of DVT under COVID-19 and bacterial pneumonia subgroups (Table 2). Of the 105 ARDS patients with COVID-19, the independent contributors to DVT were higher CK-MB levels (HR, 1.014, 95% CI: 1.005–1.024; *P* = 0.003), lower PaO_2_/FiO_2_ ratios (HR, 0.997, 95% CI: 0.993–1.000; *P* = 0.081), and D-dimer levels ≥0.5 μg/mL (HR, 2.655, 95% CI: 0.945–7.456; *P* = 0.064), whereas in the bacterial pneumonia ARDS group, DVT was independently associated with IMV (HR, 3.029, 95% CI: 1.541–5.593; *P* = 0.001) and VTE prophylaxis (HR, 0.467, 95% CI: 0.267–0.816; *P* = 0.007). Increased CK-MB levels were only independently associated with increased incidence of DVT for patients with COVID-19 (test for interaction, *P* = 0.016; Fig. 3 and Table 2), whereas VTE prophylaxis was only independently associated with lower incidence of DVT for patients with bacterial pneumonia (test for interaction, *P* = 0.022; Table 2). In addition, IMV was independently associated with increased incidence of DVT for bacterial pneumonia ARDS patients instead of COVID-19 ARDS patients; nevertheless, the interaction analysis showed no significant difference between these two groups (test for interaction, *P* = 0.372; Table 2). There was no association between serum creatinine levels and incidence of DVT in both COVID-19 ARDS group and bacterial pneumonia ARDS group, the interaction analysis displayed no significant difference between two groups (test for interaction, *P* = 0.363; Supplementary Fig. 1).

**Table 2:** Risk factors of DVT in patients with ARDS caused by COVID-19 and bacterial pneumonia

Variable	Total ARDS (N = 240)		Bacterial Pneumonia (N = 135)		COVID-19 (N = 105)		*P* for Interaction With COVID-19 Status
	Adjusted HR (95% CI)	*P* value	Adjusted HR (95% CI)	*P* value	Adjusted HR (95% CI)	*P* value	
Age, per 10 years	1.143 (0.996, 1.311)	0.056	1.164 (0.935, 1.449)	0.174	1.055 (0.861, 1.292)	0.606	0.644
Serum creatinine, per 10 μmol/L	0.956 (0.925, 0.988)	0.007	0.960 (0.913, 1.010)	0.118	0.989 (0.967, 1.012)	0.335	0.072
Serum procalcitonin, per 1 ng/L	1.003 (0.978, 1.027)	0.840	1.020 (0.986, 1.055)	0.262	1.358 (0.832, 2.217)	0.221	0.363
CK-MB, per 1 U/L	0.998 (0.993, 1.002)	0.258	0.992 (0.983, 1.002)	0.114	1.014 (1.005, 1.024)	0.003	0.016
PaO_2_/FiO_2_, per 1 mmHg	0.996 (0.993, 0.999)	0.015	0.998 (0.992, 1.003)	0.433	0.997 (0.993, 1.000)	0.081	0.480
D-dimer							
< 0.5 μg/mL	Reference		Reference		Reference		
≥ 0.5 μg/mL	2.011 (1.208, 3.347)	0.007	1.526 (0.777, 2.999)	0.220	2.655 (0.945, 7.456)	0.064	0.457
IMV							
No	Reference		Reference		Reference		
Yes	1.687 (1.140, 2.496)	0.009	3.029 (1.541, 5.953)	0.001	0.798 (0.441, 1.443)	0.455	0.372
VTE prophylaxis							
No	Reference		Reference		Reference		
Yes	0.796 (0.539, 1.175)	0.250	0.467 (0.267, 0.816)	0.007	1.367 (0.755, 2.478)	0.303	0.022

Based on univariate test results and prior knowledge, death was used as a competitive risk; Fine and Gray competing risk analysis was performed in the ARDS cohorts. The interactions of ARDS type (COVID-19 status) with age, serum creatinine level, serum procalcitonin level, CK-MB level, PaO_2_/FiO_2_, D-dimer level, and IMV, were included in the analysis

Abbreviations: *ARDS* acute respiratory distress syndrome, *CK* creatine kinase isoenzyme, *CI* confidence interval, *COVID-19* coronavirus disease 2019, *DVT* deep venous thrombosis, *FiO*_*2*_ a fraction of inspired oxygen, *IMV* invasive mechanical ventilation, *OR* odds ratio, *PaO*_*2*_ partial pressure of arterial oxygen, *VTE* venous thromboembolism

### Comparison of diagnostic accuracy for assessing the risk of DVT of different ROCs in ARDS cohort caused by COVID-19

**Fig. 4:**
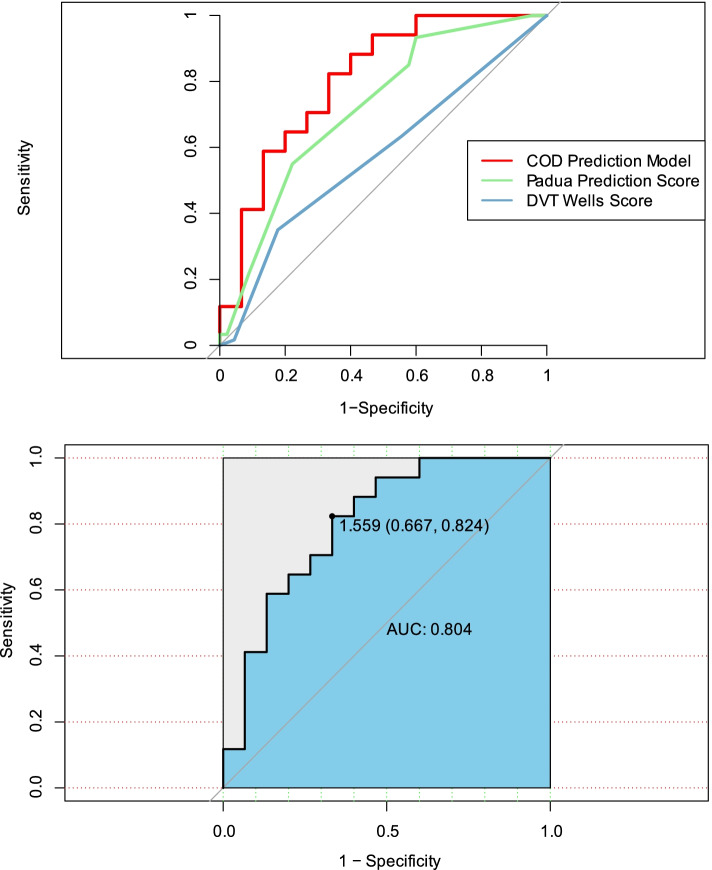
Comparison of diagnostic accuracy for screening for DVT of different ROCs in ARDS cohort caused by COVID-19. We selected the risk factors based on the test results of the Fine-Gray model and proposed a combining prediction model for assessing the risk of DVT in patients with ARDS caused by COVID-19. Patients were split by generating random numbers to produce a training data set (n*0.7) and a validation data set (n*0.3) in the ARDS cohort caused by COVID-19. The COD model including CK-MB, PaO_2_/FiO_2_ ratio, and D-dimer level shows satisfactory predicting ability for DVT (AUC = 0.803; 95% CI: 0.641–0.961; sensitivity: 66.7%; specificity: 82.4%) and was significantly higher than that of the DVT Wells score (*P* = 0.020 for these two curves); there was no significant difference compared with the Padua prediction score (*P* = 0.363 for these two curves). Abbreviations: COD = CK-MB + PaO_2_/FiO_2_ ratio + D-dimer level; ARDS, acute respiratory distress syndrome; AUC, area under the curve; CI, confidence interval; CK, creatine kinase isoenzyme; DVT, deep vein thrombosis; FiO_2_, a fraction of inspired oxygen; PaO_2_, partial pressure of arterial oxygen; ROC, receiver operating characteristic

We selected the risk factors based on the test results of the Fine-Gray model and proposed three new ways of combining forecasting models for assessing the risk of DVT in patients with ARDS caused by COVID-19 who were split by generating random numbers to produce a training data set (n*0.7) and a validation data set (n*0.3). The CK-MB level showed satisfactory predicting ability for DVT (AUC = 0.639; 95% CI: 0.428–0.850; sensitivity: 70.6%; specificity: 73.3%); yet, there was no significant difference between CK-MB level and the DVT Wells score (AUC = 0.537; *P* = 0.587 for these two curves) and the Padua prediction score (AUC = 0.717; *P* = 0.515 for these two curves; Supplementary Fig. 2) when predicting DVT. Similar results were obtained for the CO model, including CK-MB and PaO_2_/FiO_2_ ratio, which showed satisfactory predicting ability for DVT (AUC = 0.702; 95% CI: 0.516–0.887; sensitivity: 58.8%; specificity: 73.3%); however, there was no significant difference between the CO model and the DVT Wells score (*P* = 0.242 for these two curves) and the Padua prediction score (*P* = 0.888 for these two curves; Supplementary Fig. 2). However, the COD model, including CK-MB, PaO_2_/FiO_2_ ratio, and D-dimer level, showed satisfactory predicting ability for DVT (AUC = 0.803; 95% CI: 0.641–0.961; sensitivity: 66.7%; specificity: 82.4%) and better performance in predicting DVT compared to the Wells score (*P* = 0.020 for these two curves), but not compared to the Padua prediction score (*P* = 0.363 for these two curves; Fig. 4).

### Nomogram for assessing the risk of DVT

In order to increase the practicability of the prediction model, we created a nomogram based on the selected predictors (Supplementary Fig. 3). There are three prediction variables. The corresponding points were obtained by making a vertical line upward based on the value of each variable. The total points were obtained by adding the points of the three variables. The probability DVT in 5 days, 7 days, and 14 days was obtained by making a vertical line downward based on the total points. The calibration plots showed good consistency of 5-, 7-, and 14 days DVT between the actual observation and the nomogram prediction (Supplementary Fig. 4).

## Discussion

The present retrospective cohort study revealed a higher incidence of DVT on ultrasound scans in the COVID-19-associated ARDS cohort compared to the bacterial pneumonia-associated ARDS cohort (57.1% vs 41.5%, *P* = 0.016). Particularly, the incidence of proximal DVT was significantly higher in patients with COVID-19, which occurred at 3 times the rate (15.2% vs 4.4%, *P* = 0.004). Fine-Gray competing risk analysis further showed that increased CK-MB levels, decreased PaO_2_/FiO_2_ ratios, and increased D-dimer levels were independently associated with increased DVT in the COVID-19 cohort. In the bacterial pneumonia cohort, however, increased DVT was only associated with IMV, while VTE prophylaxis was associated with a lower incidence of DVT. Although it is worth noting that after taking death as a competitive risk, the Fine-Gray test showed no significant difference in the 28-day cumulative incidence of DVT between these two groups (*P* = 0.220). One reason could be the small sample size, which may reduce the power of the test.

Previous studies of critically ill patients showed that different pathogenic types might account for the high prevalence of DVT [21–23]. Meanwhile, hypercoagulability appears to be a typical feature of patients with COVID-19 [24]. Some papers pointed out that for patients with COVID − 19, especially critically ill patients, the incidence of VTE was still high, despite the fact that VTE prophylaxis was given [6–8]. However, no previous study has compared the differences in DVT between patients with ARDS caused by COVID-19 pneumonia and those caused by bacterial pneumonia. To the best of our knowledge, this study may be the earlier interpretation of differences in DVT in patients with ARDS caused by different pathogens.

Several reasons may account for the notably higher incidence of DVT in the COVID-19 pneumonia patients with ARDS. First, the coagulation pathway can be activated through the contact system and kallikrein/kinin system (KKS) [25]. Studies have discovered that KKS is dysregulated when SARS-CoV-2 binds to the angiotensin-converting enzyme II (ACE-2) receptor of vascular endothelium, which may be a more reasonable mechanism for the noted interaction between COVID-19 and DVT [26, 27]. Second, high plasma levels of proinflammatory cytokines were observed in the severe COVID-19 [28]. The direct activation of the coagulation cascade by a cytokine storm is conceivable. Third, the immune-mediated damage according to the acute coronavirus infections may partially contribute to DVT [29]. Finally, clinicians in clinical practice found that approximately 20% of COVID-19 patients had severe coagulation abnormalities, and almost all the patients with severe and critically ill SARS-CoV-2 infection showed major coagulation disorders [30, 31]. Compared with the bacterial pneumonia cohort, patients in the COVID-19 cohort had higher D-dimer levels and longer APTT, which is consistent with previous studies.

A significant interaction term indicated that CK-MB levels had a different effect in the two groups. The incidence of DVT in the COVID-19 pneumonia patients with ARDS increased with the raising of the CK-MB levels. Aside from the lacking knowledge on its pathophysiology, the main proposed mechanisms are as follows: heart and arterial vascular system injury occur due to increased oxygen demand but in the context of hypoxemia triggered by cytokine storm and systemic immune response, which are most frequently encountered among patients with COVID-19 cases [32–37]. Likewise, it has been hypothesized that direct viral toxicity through the interaction with ACE-2 receptors is highly expressed by some pericytes [34]. So, considering the comprehensively above-mentioned factors, it was found that severe COVID-19 cases had elevated levels of biomarkers of cardiovascular system injury, such as CK-MB. As a marker of myocardial damage, elevated CKMB levels may be associated with the severity of COVID-19 in critically ill patients. Patients with severe disease may have increased CK-MB levels and more sever inflammatory response, which place them at higher risk of developing DVT. However, whether there is a direct causal relationship between CK-MB levels and DVT needs to be further investigated.

Therefore, a thorough assessment should be conducted in the follow-up of severe COVID-19 patients with ARDS, and adequate measures should be managed to detect, diagnose, and treat VTE at their early stage, considering the high-risk of developing DVT**.**

VTE prevention is commonly applied in patients with COVID-19 [3–5, 9]. However, some studies suggested that VTE prophylaxis may not be effective enough in preventing DVT events for critically ill COVID-19 patients [6–8]. In this study, Fine-Gray competing risk analysis showed that VTE prophylaxis was effective in preventing DVT in patients with bacterial pneumonia-associated ARDS, while there was no significant association between VTE prophylaxis and DVT events in COVID-19 patients with ARDS, which suggests that COVID-19 patients may have more severe hypercoagulability and higher risk of VTE.

Fine-Gray competing risk analysis showed no association between serum creatinine levels and incidence of DVT in both COVID-19 ARDS group and bacterial pneumonia ARDS group and no significant difference between the two groups by the interaction analysis (test for interaction, *P* = 0.363). Yet, some studies have demonstrated that renal impairment is an independent risk factor for DVT [38, 39]. It is worth noting that other studies have shown that LWMH may have different levels of bioaccumulation in the case of renal insufficiency [40, 41]. So, we speculate that the same dose of LWMH may play a stronger role in preventing DVT because of renal insufficiency. Cook et al. indicated that the incidence of DVT for patients with renal insufficiency in an intensive care unit (ICU) who received dalteparin 5000 IU once daily was 5.1% [42], which was far lower than that in the overall population of critically ill patients who received prophylaxis recommended by the guidelines [43, 44]. Based on the speculation that renal function impairment leads to decreased metabolism of LMWH, our previous study [10] showed that the increased serum creatinine levels in ARDS patients with bacterial pneumonia might be a protective factor for DVT; Yet, VTE prophylaxis was not included in the multivariate model of that study. In the present study, we enrolled VTE prophylaxis, which is an important factor for VTE in the Fine-Gray competing risk analysis, and found that VTE prophylaxis is a protective factor for DVT in patients with bacterial pneumonia rather than an elevated creatinine level alone. Still, the limited protective effect of VTE prophylaxis against VTE events in COVID-19 patients remains an issue to be resolved.

Multivariable analysis showed an association only among CK-MB levels, PaO_2_/FiO_2_ ratios, D-dimer levels ≥0.5 μg/mL, and DVT in the COVID-19 cohort. Using a ROC analysis, a combination of these corresponding indicators yielded a sensitivity of 66.7% and a specificity of 82.4% when predicting DVT in COVID-19 patients with ARDS, and the AUC-ROC was 0.804. The statistical test showed that the prediction power of this model was significantly better than the DVT Wells score and had no significant difference compared with the Padua prediction score. Next, a combined prediction model was identified to effectively depict prediction for DVT in this group by drawing a nomogram and its calibration curve. A possible reason for the superiority of this new prediction model is that the commonly used predictive scoring systems such as the Padua score and Wells score apply to the general medical and surgical patients in the hospital. As a serious clinical pathophysiological syndrome with an overwhelming inflammatory response and coagulation abnormalities, ARDS caused by COVID-19 has unique clinical characteristics and serious complications.

Similar to some previous studies [9, 10], our data suggested that DVT is associated with adverse outcomes. The 28-day mortality was significantly higher in both COVID − 19 and bacterial pneumonia groups. The worse outcome in the COVID-19 cohort may result from the inflammatory response to SARS-CoV-2 infection resulting in thrombo-inflammation and driving thrombosis [45]. Coagulation activation could also be associated with a sustained inflammatory response [46]. In addition, there is a 50% chance for patients with untreated proximal DVT to develop symptomatic PE within 3 months [47]. PE might aggravate the hypoxemia of ARDS patients and then result in lower actuarial survival rates. If there is any clinical suspicion of PE, a CTPA should be considered and obtained. Unfortunately, due to the critical condition of ARDS patients, CTPA examination was limited. CTPA examination was performed only for one COVID-19 patient who was highly suspected of PE and consequently diagnosed with PE. By contrast, in the bacterial pneumonia cohort, we performed 5 CTPA examinations, and 3 patients were diagnosed with PE. Using the figures given above, we may underestimate the incidence of PE. The potential presence of PE associated with DVT may be associated with poor survival in patients with DVT. Although these findings were not surprising, given that our patient population was composed of older severely ill patients at high risk for DVT with other organ-related diseases, our data raised the question of screening for DVT, risk stratification, and potential VTE prophylaxis to improve outcomes in ARDS patients infected with COVID-19 and those infected with bacterial pneumonia.

This study has some limitations. First, this was a retrospective study that included data from two independent single-center cohorts, which may have resulted in selection bias. Second, the study has a small sample size. Third, due to the critical condition of patients with ARDS, CTPA examinations were restricted, which significantly underestimated the incidence of PE. Fourth, although there was no difference in PaO_2_/FiO_2_ between the two groups, patients with COVID-19 had a lower proportion of IMV and lower severity of illness (lower APACHE II score and SOFA score), which may have reduced the difference in DVT between the two groups. The trend towards a higher incidence of DVT in COVID-19 patients might have been more pronounced if the two groups had been more matched in terms of severity of illness (baseline comorbidities). Finally, the data from the bacterial pneumonia cohort originated from a 6-year span, whereas the data from the COVID-19 cohort originated from only a 1-month span, which may also have affected the study’s results.

## Conclusions

Compared with patients with ARDS caused by bacterial pneumonia, the incidence of DVT is higher in patients with ARDS caused by COVID-19, and the risk factors for DVT are completely different. Also, a prediction model based on the combination of CK-MB levels, PaO_2_/FiO_2_ ratios, and D-dimer levels has been identified effectively for assessing risk of DVT in ARDS patients with COVID-19. It is suggested that COVID-19 is probably an additional risk factor for DVT in ARDS patients. Therefore, future studies investigating the correlation between DVT and COVID-19 should focus on the COVID-19 and its implications for thrombosis and anticoagulation, which could provide more experience and evidence regarding COVID-19 treatment measures.

## Supplementary Information

**Additional file 1.**

**Additional file 2.**

**Additional file 3.**

**Additional file 4.**

**Additional file 5.**

## Data Availability

All data analyzed during the study are presented in the main manuscript. In addition, the anonymous dataset is available from the corresponding author upon reasonable request.

## Abbreviations

ACE-2: Angiotensin-converting enzyme II
ALT: Alanine aminotransferase
APACHE: Acute Physiology and Chronic Health Evaluation
ARDS: Acute respiratory distress syndrome
AST: Aspartate aminotransferase
AUC: Area under the curve
BUN: Blood urea nitrogen
CI: Confidence interval
CK: Creatine kinase isoenzyme
COVID-19: Coronavirus disease 2019
CTPA: Computed tomography pulmonary angiography
CVC: Central venous catheterization
DBIL: Direct bilirubin
DVT: Deep vein thrombosis
eGFR: Estimated glomerular filtration rate
FiO_2_: Fraction of inspired oxygen
IMV: Invasive mechanical ventilation
IQR: Interquartile range
KKS: Kallikrein/kinin system
LDH: Lactate dehydrogenase
MV: Mechanical ventilation
OR: Odds ratio
PaO_2_: Partial pressure of arterial oxygen
PCT: Procalcitonin
PE: Pulmonary embolism
PT: Prothrombin time
ROC: Receiver operating characteristic
SARS-CoV-2: Severe acute respiratory syndrome coronavirus 2
SD: Standard deviation
SOFA: SEQUENTIAL Organ Failure Assessment
TBIL: Lower total bilirubin
VTE: Venous thromboembolism
WBC: White blood cell
