# Supplementary figures and images for: Comparison of deep vein thrombosis risks in acute respiratory distress syndrome caused by COVID-19 and bacterial pneumonia: a retrospective cohort study
Source: Thromb J. 2022 May 10;20:27. doi: 10.1186/s12959-022-00386-y (PMC9086137; doi:10.1186/s12959-022-00386-y)

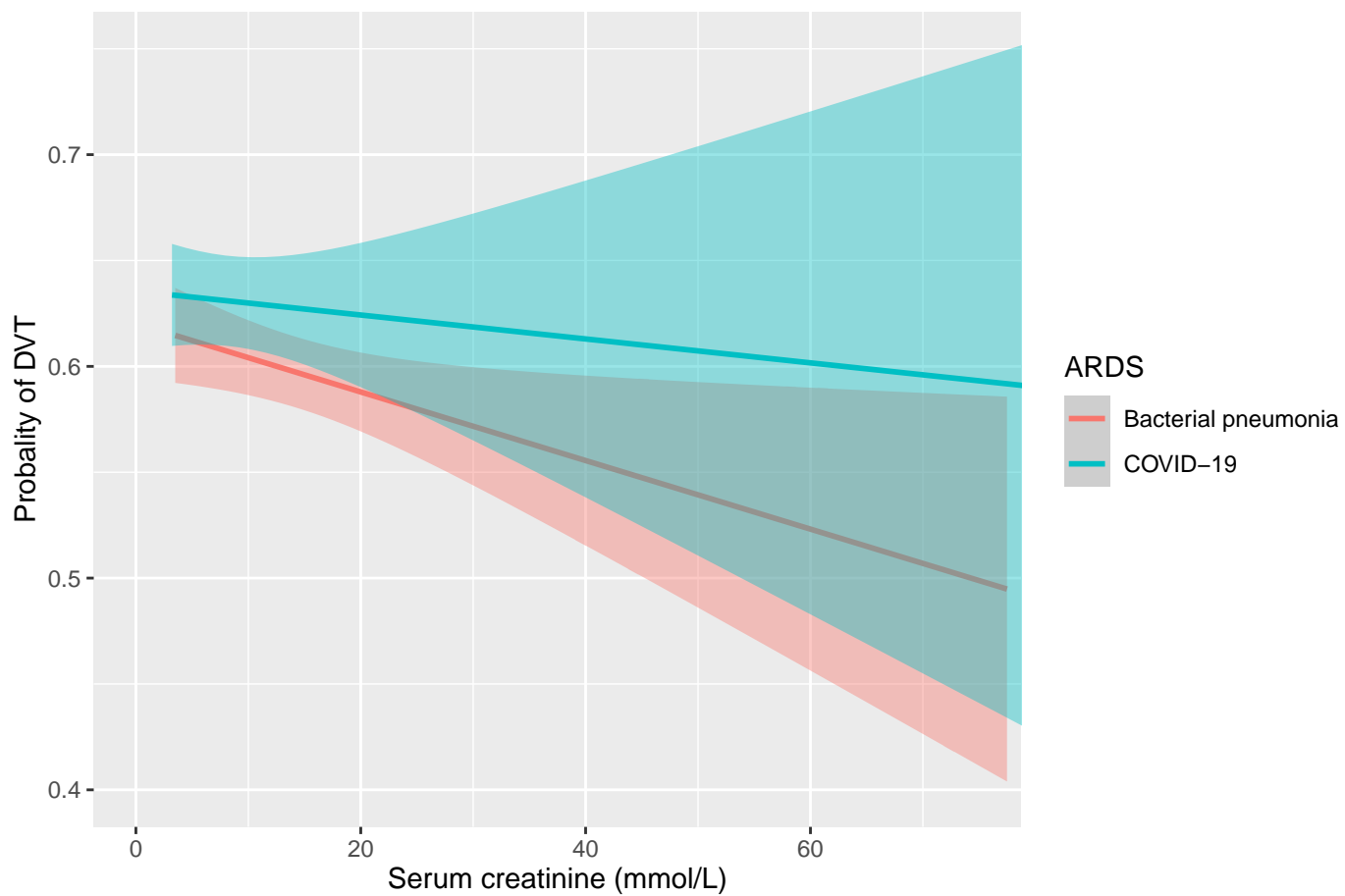

Supplement: Supplementary file 1 — Additional file 1. [file 12959_2022_386_MOESM1_ESM.pdf]

A

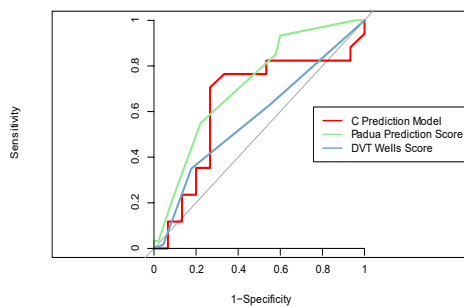

B

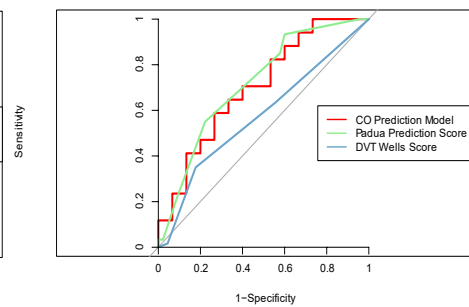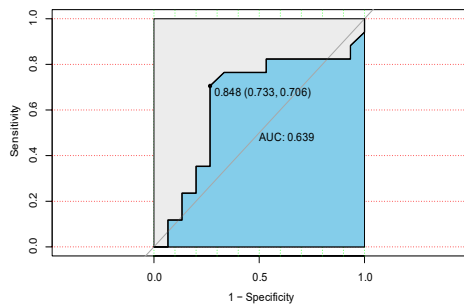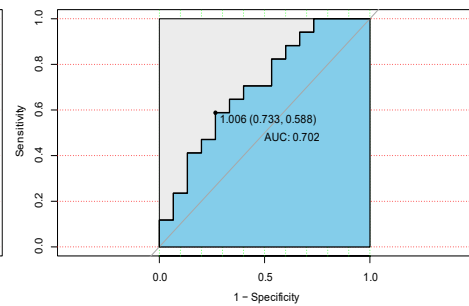

Supplement: Supplementary file 2 — Additional file 2. [file 12959_2022_386_MOESM2_ESM.pdf]

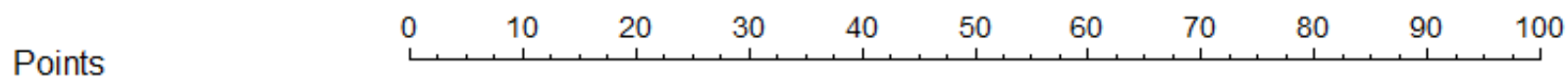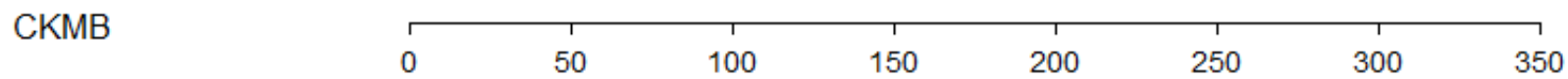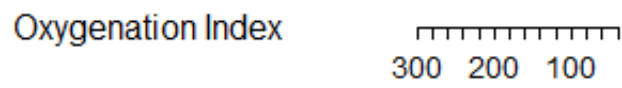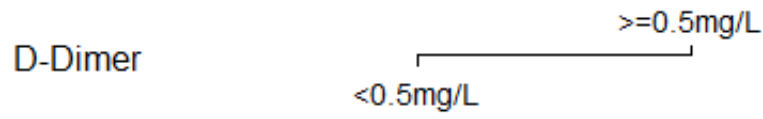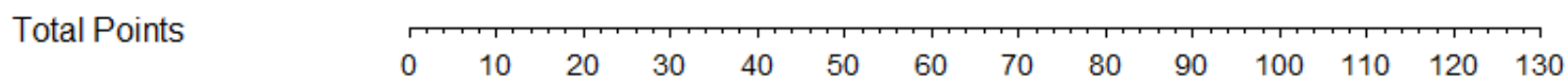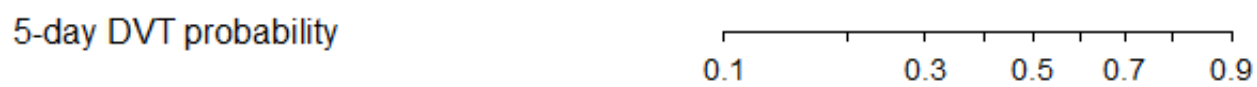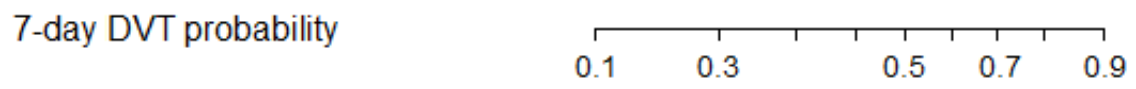

Supplement: Supplementary file 3 — Additional file 3. [file 12959_2022_386_MOESM3_ESM.pdf]

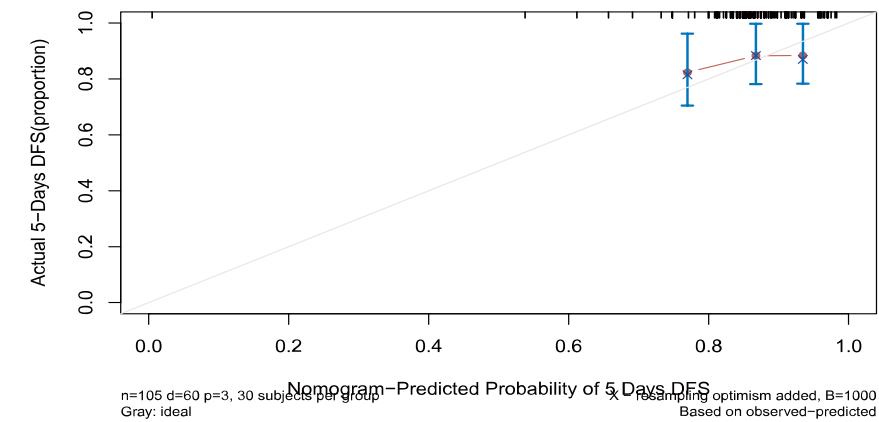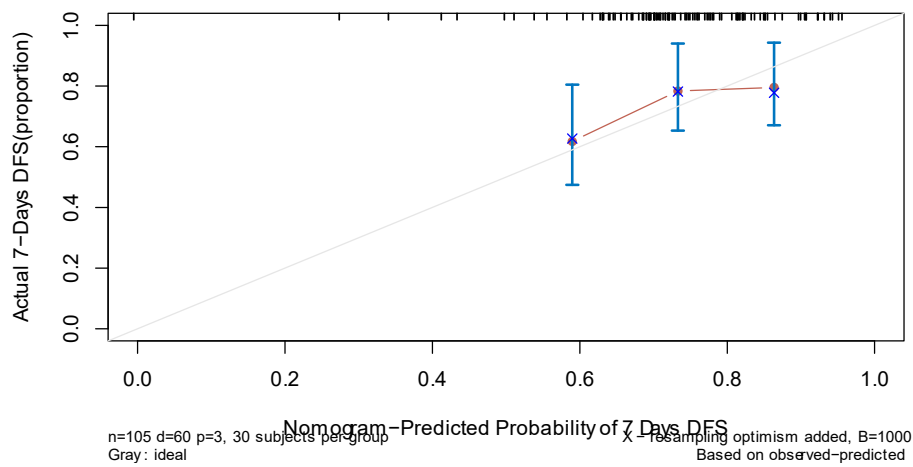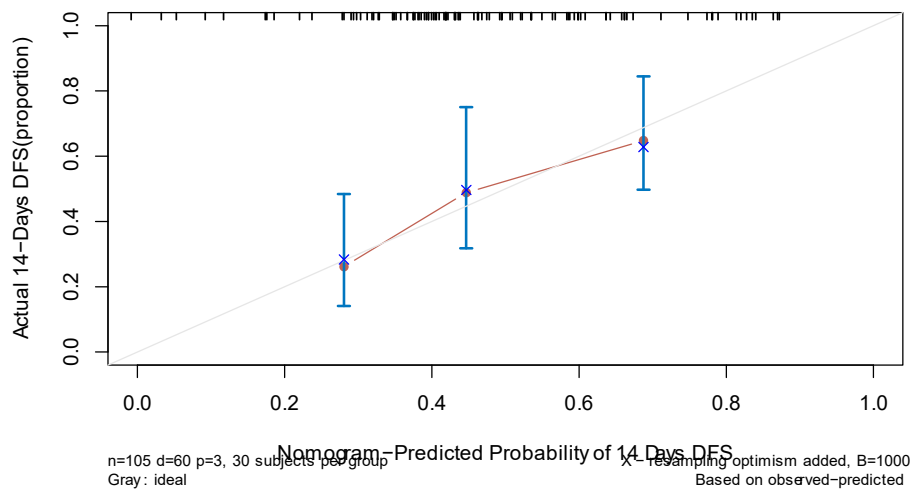

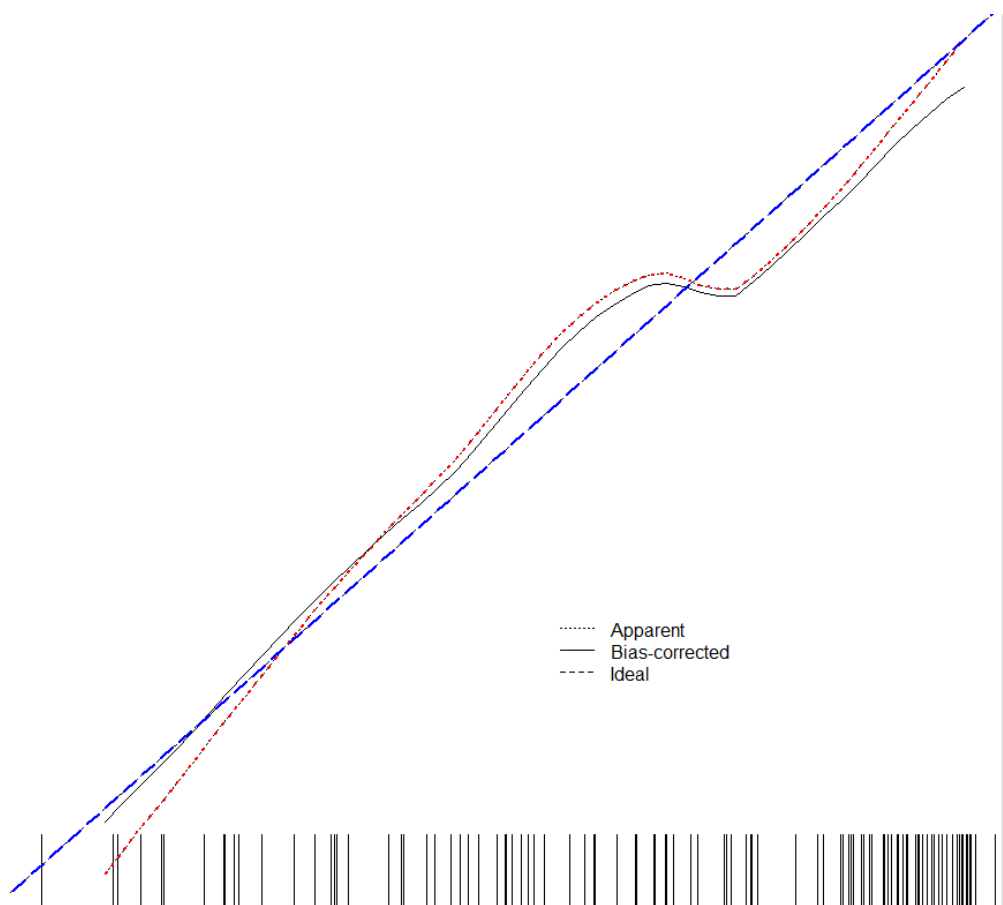

Calibration curve for nomogram prediction

Supplement: Supplementary file 4 — Additional file 4. [file 12959_2022_386_MOESM4_ESM.pdf]
