# Supplementary material for: Comparison of deep vein thrombosis risks in acute respiratory distress syndrome caused by COVID-19 and bacterial pneumonia: a retrospective cohort study
Source: Thromb J. 2022 May 10;20:27. doi: 10.1186/s12959-022-00386-y (PMC9086137; doi:10.1186/s12959-022-00386-y)
Supplement: Supplementary file 5 — Additional file 5. [file 12959_2022_386_MOESM5_ESM.docx]

**Supplementary Table 1 Demographic and clinical characteristics of DVT vs Non-DVT patients in overall ARDS**

| **Characteristic** | **Total**  **(N = 240)** | **Non-DVT**  **(N = 124)** | **DVT**  **(N = 116)** | ***P* value** |
| --- | --- | --- | --- | --- |
| Age, y | 64.3 ± 14.2 | 61.5 ± 16.1 | 67.2 ± 11.2 | 0.002 |
| Male | 161 (67.1) | 86 (69.4) | 75 (64.7) | 0.439 |
| BMI, kg/m^2^ | 23.6 ± 3.4 | 23.7±3.8 | 23.5±3.0 | 0.605 |
| Bed time ≥ 3 days | 189 (78.8) | 90 (72.6) | 99 (85.3) | 0.016 |
| Hospital stays, d | 23 (13, 38) | 22 (12, 38) | 23 (13, 38) | 0.792 |
| ARDS to DVT or last US scan, d | 7 (3, 13) | 8 (3, 14) | 7 (4, 12) | 0.725 |
| Median number of US scans | 1 (1, 2) | 1 (1, 2) | 1 (1, 2) | 0.904 |
| DVT Wells score | 1 (1, 2) | 1 (1, 1) | 1 (1, 2) | 0.623 |
| Padua prediction score | 5 (5, 6) | 5 (5, 6) | 6 (5, 6) | 0.003 |
| APACHE II score | 16 (12, 23) | 17 (12, 23) | 16 (11, 23) | 0.712 |
| SOFA score | 5 (4, 10) | 5 (3, 9) | 6 (4, 12) | 0.009 |
| Underlying disease | | | | |
| Smoke | 84 (35.0) | 50 (40.3) | 34 (29.3) | 0.074 |
| Chronic respiratory disease | 32 (13.3) | 12 (9.7) | 20 (17.2) | 0.085 |
| Hypertension | 103 (42.9) | 48 (38.7) | 55 (47.4) | 0.173 |
| Coronary heart disease | 39 (16.3) | 17 (13.7) | 22 (19.0) | 0.270 |
| Diabetes | 54 (22.5) | 28 (22.6) | 26 (22.4) | 0.975 |
| Cerebral vascular disease | 31 (12.9) | 18 (14.5) | 13 (11.2) | 0.445 |
| Chronic liver disease | 5 (2.1) | 4 (3.2) | 1 (0.9) | 0.371 |
| Chronic kidney disease | 16 (6.7) | 10 (8.1) | 6 (5.2) | 0.369 |
| Symptoms | | | | |
| Fever | 222 (92.5) | 114 (91.9) | 108 (93.1) | 0.731 |
| Cough | 188 (78.3) | 100 (80.6) | 88 (75.9) | 0.369 |
| Dyspnea | 198 (82.5) | 104 (83.9) | 94 (81.0) | 0.563 |
| DVT symptoms | 46 (19.2) | 24 (19.4) | 22 (19.0) | 0.939 |
| Edema of lower extremities | 42 (17.5) | 24 (19.4) | 18 (15.5) | 0.434 |
| Leg pain | 6 (2.5) | 0 (0.0) | 6 (5.2) | 0.012 |
| Arterial blood gas analysis | | | | |
| PaO_2_/FiO_2_, mm Hg | 135 (81, 195) | 158 (97, 212) | 100 (73, 168) | < 0.001 |
| Hematologic and infection-related indices | | | | |
| White blood cell count,  ×10^9^/L | 10.9 (7.23, 16.0) | 11.2 (6.7, 16.8) | 10.9 (7.6, 15.6) | 0.906 |
| Neutrophil count, ×10^9^/L | 9.7 (5.9, 14.2) | 9.9 (5.1, 14.1) | 9.6 (6.2, 14.5) | 0.804 |
| Lymphocyte count, ×10^9^/L | 0.8 (0.5, 1.2) | 0.8 (0.5, 1.2) | 0.7 (0.5, 1.1) | 0.157 |
| Neutrophil-to-lymphocyte ratio | 12.5 (7.1, 21.0) | 11.8 (6.3, 19.4) | 13.7 (7.9, 21.6) | 0.137 |
| Platelet count, ×10^9^/L | 190 (133, 263) | 193 (129, 262) | 187 (135, 266) | 0.740 |
| Hemoglobin, g/L | 116 (99, 130) | 116 (95, 135) | 115 (102, 127) | 0.633 |
| C-reactive protein, mg/L | 99.5 (51.8, 120.0) | 110.4 (51.3, 120.0) | 96.8 (52.7, 120.0) | 0.550 |
| Serum procalcitonin, ng/L | 0.8 (0.1, 4.2) | 0.8 (0.1, 3.7) | 0.8 (0.2, 4.6) | 0.550 |
| Biochemical test | | | | |
| Total protein, g/L | 57.6 (50.9, 63.2) | 57.6 (51.5, 63.0) | 57.6 (50.1, 64.2) | 0.950 |
| Albumin, g/L | 26.3 (23.6, 29.9) | 26.9 (23.8, 30.4) | 26.2 (23.3, 28.5) | 0.076 |
| Aspartate aminotransferase,  U/L | 35.0 (25.5, 58.0) | 35.0 (23.6, 59.5) | 35.0 (27.0, 58.0) | 0.736 |
| Alanine aminotransferase,  U/L | 33.2 (19.6, 60.2) | 32.0 (18.6, 54.5) | 33.3 (22.7, 62.5) | 0.207 |
| Total bilirubin, μmol/L | 14.2 (10.1, 20.3) | 13.6 (9.7, 19.5) | 14.7 (10.8, 20.5) | 0.256 |
| Direct bilirubin, μmol/L | 4.8 (3.1, 7.3) | 4.4 (2.7, 7.6) | 4.9 (3.6, 7.1) | 0.050 |
| Lactate dehydrogenase, U/L | 354.0 (234.3, 546.0) | 314.5 (212.6, 443.5) | 391.5 (267.0, 611.6) | 0.003 |
| Blood urea nitrogen, mmol/L | 7.54 (4.80, 13.78) | 6.88 (4.65, 15.23) | 7.72 (5.25, 12.96) | 0.365 |
| Serum creatinine, μmol/L | 73.5 (56.7, 125.8) | 78.1 (57.7, 172.0) | 72.8 (56.4, 98.8) | 0.049 |
| eGFR, mL/min/1.73m^2^ | 88.3 (44.2, 104.2) | 86.4 (32.7, 108.3) | 88.7 (55.5, 102.5) | 0.602 |
| Creatine kinase isoenzyme  MB, U/L | 16.2 (10.8, 29.7) | 15.3 (10.3, 28.0) | 17.0 (11.8, 30.5) | 0.147 |
| Coagulation function | | | | |
| D-dimer, µg/mL | 1.8 (0.7, 4.6) | 1.1 (0.5, 2.3) | 2.7 (1.1, 8.0) | < 0.001 |
| Prothrombin time, s | 13.6 (12.60, 15.1) | 13.4 (12.2, 15.0) | 13.8 (12.7, 15.1) | 0.025 |
| Activated partial  thromboplastin time, s | 33.7 (29.7, 38.1) | 33.7 (29.7, 37.7) | 33.7 (29.4, 38.7) | 0.984 |
| Treatments | | | | |
| Glucocorticoid therapy | 90 (37.5) | 38 (30.6) | 52 (44.8) | 0.023 |
| Immunoglobulin therapy | 56 (23.3) | 20 (16.1) | 36 (31.0) | 0.006 |
| CVC | 82 (34.2) | 37 (29.8) | 45 (38.8) | 0.144 |
| CRRT | 22 (9.2) | 12 (9.7) | 10 (8.6) | 0.777 |
| IMV | 103 (42.9) | 40 (32.3) | 63 (54.3) | 0.001 |
| Sedative therapy | 86 (35.8) | 33 (26.6) | 53 (45.7) | 0.002 |
| Vasoactive drugs | 64 (26.7) | 26 (21.0) | 38 (32.8) | 0.039 |
| VTE prophylaxis | 137 (57.1) | 69 (55.6) | 68 (58.6) | 0.642 |
| LMWH | 117 (48.8) | 54 (43.5) | 63 (54.3) | 0.096 |
| LMWH + physical | 80 (33.3) | 38 (30.6) | 42 (36.2) | 0.361 |
| Physical prophylaxis only | 19 (7.9) | 15 (12.1) | 4 (3.4) | 0.013 |
| 28-day mortality | 73 (30.4) | 24 (19.4) | 49 (42.2) | < 0.001 |

Data are presented as mean ± SD, median (IQR), or n (%). *P* values comparing DVT and non-DVT groups were from a two-sample *t-*test, Mann-Whitney *U* test, χ² test, or Fisher exact test. *P* < 0.05 was considered statistically significant.

Abbreviations: APACHE, Acute Physiology and Chronic Health Evaluation; ARDS, acute respiratory distress syndrome; BMI, body mass index; CK, creatine kinase isoenzyme; COVID-19, coronavirus disease 2019; CRRT, continuous renal replacement therapy; CVC, central venous catheterization; DVT, deep venous thrombosis; eGFR, estimated glomerular filtration rate; FiO_2_, a fraction of inspired oxygen; IMV, invasive mechanical ventilation; IQR, interquartile range; LMWH, low molecular weight heparin; PaO_2_, partial pressure of arterial oxygen; SD, standard deviation; SOFA, Sequential Organ Failure Assessment; US, ultrasound; VTE, venous thromboembolism.

**Supplementary Table 2 Demographic and clinical characteristics of DVT vs Non-DVT in patients with ARDS caused by COVID-19**

| **Characteristic** | **COVID-19**  **(N = 105)** | **Non-DVT**  **(n = 45)** | **DVT**  **(n = 60)** | ***P* value** |
| --- | --- | --- | --- | --- |
| Age, y | 63.6 ± 13.1 | 60.2 ± 14.5 | 66.0 ± 11.4 | 0.031 |
| Male | 60 (57.1) | 25 (55.6) | 35 (58.3) | 0.776 |
| BMI, kg/m^2^ | 23.6 ± 2.7 | 23.2 ± 3.0 | 23.8 ± 2.4 | 0.263 |
| Bed time ≥ 3 days | 72 (68.6) | 24 (53.3) | 48 (80.0) | 0.004 |
| Hospital stays, d | 31 (18, 41) | 34 (20, 41) | 29 (16, 43) | 0.600 |
| ARDS to DVT or last US scan, d | 10 (6, 13) | 10 (8, 15) | 9 (6, 13) | 0.305 |
| Median number of US scans | 1 (1, 1) | 1 (1, 1) | 1 (1, 1) | 0.188 |
| DVT Wells score | 1 (0, 2) | 1 (0, 1) | 1 (0, 2) | 0.172 |
| Padua prediction score | 5 (4, 6) | 5 (2, 5) | 6 (5, 6) | <0.001 |
| APACHE II score | 11 (11, 13) | 11 (10, 13) | 12 (10, 15) | 0.130 |
| SOFA score | 4 (3, 12) | 3 (3, 6) | 5 (4, 13.5) | <0.001 |
| Underlying disease | | | | |
| Smoke | 8 (7.6) | 3 (6.7) | 5 (8.3) | 0.750 |
| Chronic respiratory disease | 7 (6.7) | 2 (4.4) | 5 (8.3) | 0.429 |
| Hypertension | 43 (41.0) | 15 (33.3) | 28 (46.7) | 0.169 |
| Coronary heart disease | 15 (14.3) | 6 (13.3) | 9 (15.0) | 0.809 |
| Diabetes | 21 (20.0) | 9 (20.0) | 12 (20.0) | 1.000 |
| Cerebral vascular disease | 4 (3.8) | 2 (4.4) | 2 (3.3) | 0.766 |
| Chronic liver disease | 3 (2.9) | 2 (4.4) | 1 (1.7) | 0.398 |
| Chronic kidney disease | 3 (2.9) | 2 (4.4) | 1 (1.7) | 0.398 |
| Symptoms | | | | |
| Fever | 94 (89.5) | 41 (91.1) | 53 (88.3) | 0.646 |
| Cough | 74 (70.5) | 34 (75.6) | 40 (66.7) | 0.323 |
| Dyspnea | 68 (64.7) | 29 (64.4) | 39 (65.0) | 0.953 |
| DVT symptoms | 19 (18.1) | 7 (15.6) | 12 (20.0) | 0.558 |
| Edema of lower extremities | 15 (14.3) | 7 (15.6) | 8 (13.3) | 0.747 |
| Leg pain | 4 (3.8) | 0 (0.0) | 8 (13.3) | 0.077 |
| Arterial blood gas analysis | | | | |
| PaO_2_/FiO_2_, mm Hg | 130 (81, 197) | 160 (100, 224) | 100 (77, 174) | 0.003 |
| Hematologic and infection-related indices | | | | |
| White blood cell count,  ×10^9^/L | 8.1 (5.7, 10.9) | 6.9 (4.8, 10.1) | 8.9 (6.9, 10.9) | 0.008 |
| Neutrophil count, ×10^9^/L | 6.5 (4.2, 9.6) | 5.3 (3.7, 8.7) | 7.3 (5.8, 9.9) | 0.007 |
| Lymphocyte count, ×10^9^/L | 0.8 (0.5, 1.1) | 0.9 (0.6, 1.1) | 0.7 (0.5, 1.1) | 0.129 |
| Neutrophil-to-lymphocyte ratio | 8.9 (4.7, 15.1) | 6.7 (3.8, 11.5) | 11.3 (6.2, 18.3) | 0.005 |
| Platelet count, ×10^9^/L | 190 (144, 270) | 218 (145, 273) | 180 (142, 266) | 0.246 |
| Hemoglobin, g/L | 118 (108, 132) | 118 (110, 132) | 118 (105, 131) | 0.727 |
| C-reactive protein, mg/L | 58.0 (20.7, 99.5) | 49.1 (10.6, 88.8) | 65.7 (29.5, 99.7) | 0.130 |
| Serum procalcitonin, ng/L | 0.1 (0.1, 0.4) | 0.1 (0.1, 0.3) | 0.2 (0.1, 0.4) | 0.001 |
| Biochemical test | | | | |
| Total protein, g/L | 60.8 (57.6, 65.3) | 60.8 (57.6, 64.8) | 60.4 (57.6, 66.3) | 0.826 |
| Albumin, g/L | 27.3 (24.2, 30.2) | 28.2 (24.8, 30.4) | 26.4 (24.1, 29.3) | 0.148 |
| Aspartate aminotransferase,  U/L | 33.0 (24.0, 44.0) | 30.0 (22.0, 36.0) | 35.0 (29.0, 49.5) | 0.011 |
| Alanine aminotransferase,  U/L | 35.0 (26.0, 62.0) | 32.0 (19.0, 46.0) | 37.5 (31.0, 64.0) | 0.015 |
| Total bilirubin, μmol/L | 13.6 (9.2, 17.0) | 11.3 (8.3, 15.3) | 14.3 (10.5, 17.5) | 0.027 |
| Direct bilirubin, μmol/L | 4.5 (3.0, 6.2) | 3.5 (2.6, 5.3) | 4.8 (3.5, 6.6) | 0.005 |
| Lactate dehydrogenase, U/L | 354.0 (224.0, 511.0) | 302.0 (199.0, 396.0) | 384.0 (306.0, 583.5) | 0.001 |
| Blood urea nitrogen, mmol/L | 6.50 (4.21, 9.21) | 5.21 (3.58, 6.92) | 7.54 (5.03, 9.96) | 0.002 |
| Serum creatinine, μmol/L | 64.3 (53.7, 75.5) | 60.5 (51.9, 71.8) | 67.3 (55.95, 76.2) | 0.173 |
| eGFR, mL/min/1.73m^2^ | 95.6 (80.8, 106.9) | 99.5 (85.3, 110.3) | 93.2 (75.1, 103.0) | 0.121 |
| Creatine kinase isoenzyme  MB, U/L | 16.2 (10.0, 31.0) | 12.0 (9.0, 16.2) | 25.5 (13.5, 35.5) | 0.001 |
| Coagulation function | | | | |
| D-dimer, µg/mL | 2.8 (1.1, 8.0) | 1.5 (0.5, 3.0) | 6.4 (1.8, 8.0) | <0.001 |
| Prothrombin time, s | 13.6 (12.7, 14.9) | 13.1 (12.4, 14.0) | 14.1 (13.4, 15.1) | 0.001 |
| Activated partial  thromboplastin time, s | 34.8 (32.5, 39.2) | 34.4 (32.6, 38.0) | 34.8 (31.2, 40.1) | 0.938 |
| Treatments | | | | |
| Glucocorticoid therapy | 50 (47.6) | 19 (42.2) | 31 (51.7) | 0.338 |
| Immunoglobulin therapy | 53 (50.5) | 18 (40.0) | 35 (58.3) | 0.063 |
| CVC | 37 (35.2) | 11 (24.4) | 26 (43.3) | 0.045 |
| CRRT | 10 (9.5) | 3 (6.7) | 7 (11.7) | 0.388 |
| IMV | 24 (22.9) | 5 (11.1) | 19 (31.7) | 0.013 |
| Sedative therapy | 24 (22.9) | 5 (11.1) | 19 (31.7) | 0.013 |
| Vasoactive drugs | 37 (35.2) | 11 (24.4) | 26 (43.3) | 0.045 |
| VTE prophylaxis | 73 (69.5) | 28 (62.2) | 45 (75.0) | 0.159 |
| LMWH | 62 (59.0) | 17 (37.8) | 45 (75.0) | < 0.001 |
| LMWH + physical | 40 (38.1) | 11 (24.4) | 29 (48.3) | 0.013 |
| Physical prophylaxis only | 11 (10.5) | 11 (24.4%) | 0 (0) | < 0.001 |
| 28-day mortality | 27 (25.7) | 7 (15.6) | 20 (33.3) | 0.039 |

Data are presented as mean ± SD, median (IQR), or n (%). *P* values comparing DVT and non-DVT groups were from a two-sample *t-*test, Mann-Whitney *U* test, χ² test, or Fisher exact test. *P* < 0.05 was considered statistically significant.

Abbreviations: APACHE, Acute Physiology and Chronic Health Evaluation; ARDS, acute respiratory distress syndrome; BMI, body mass index; CK, creatine kinase isoenzyme; COVID-19, coronavirus disease 2019; CRRT, continuous renal replacement therapy; CVC, central venous catheterization; DVT, deep venous thrombosis; eGFR, estimated glomerular filtration rate; FiO_2_, a fraction of inspired oxygen; IMV, invasive mechanical ventilation; IQR, interquartile range; LMWH, low molecular weight heparin; PaO_2_, partial pressure of arterial oxygen; SD, standard deviation; SOFA, Sequential Organ Failure Assessment; US, ultrasound; VTE, venous thromboembolism.

**Supplementary Table 3 Demographic and clinical characteristics of DVT vs Non-DVT in patients with ARDS caused by bacterial pneumonia**

| **Characteristic** | **Bacterial pneumonia**  **(N = 135)** | **Non-DVT**  **(n = 79)** | **DVT**  **(n = 56)** | ***P* value** |
| --- | --- | --- | --- | --- |
| Age, y | 64.8 ± 15.1 | 62.1 ±17.1 | 68.5 ± 11.0 | 0.060 |
| Male | 101 (74.8) | 61 (77.2) | 40 (71.4) | 0.445 |
| BMI, kg/m^2^ | 23.7 ± 3.9 | 24.0 ± 4.1 | 23.1 ± 3.5 | 0.192 |
| Bed time ≥ 3 days | 117 (86.7) | 66 (83.5) | 51 (91.1) | 0.205 |
| Hospital stays, d | 18 (11, 29) | 18 (11, 31) | 17 (9, 28) | 0.397 |
| ARDS to DVT or last US scan, d | 5 (2, 12) | 6 (2, 13) | 5 (3, 10) | 0.698 |
| Median number of US scans | 1 (1, 2) | 1 (1, 2) | 1 (1, 2) | 0.908 |
| DVT Wells score | 1 (1, 1) | 1 (1, 2) | 1 (1, 1) | 0.707 |
| Padua prediction score | 5 (5, 6) | 5 (5, 6) | 5.5 (5, 6) | 0.533 |
| APACHE II score | 22 (17, 27) | 22 (17, 27) | 23 (20, 29) | 0.150 |
| SOFA score | 6 (4, 10) | 6 (4, 10) | 6 (5, 10) | 0.544 |
| Underlying disease | | | | |
| Smoke | 76 (56.3) | 47 (59.5) | 29 (51.8) | 0.374 |
| Chronic respiratory disease | 25 (18.5) | 10 (12.7) | 15 (26.8) | 0.037 |
| Hypertension | 60 (44.4) | 33 (41.8) | 27 (48.2) | 0.458 |
| Coronary heart disease | 24 (17.8) | 11 (13.9) | 13 (23.2) | 0.164 |
| Diabetes | 33 (24.4) | 19 (24.1) | 14 (25.0) | 0.899 |
| Cerebral vascular disease | 27 (20.0) | 16 (20.3) | 11 (19.6) | 0.930 |
| Chronic liver disease | 2 (1.5) | 2 (2.5) | 0 (0.0) | 0.230 |
| Chronic kidney disease | 13 (9.6) | 8 (10.1) | 5 (8.9) | 0.816 |
| Symptoms | | | | |
| Fever | 128 (94.8) | 73 (92.4) | 55 (98.2) | 0.134 |
| Cough | 114 (84.4) | 66 (83.5) | 48 (85.7) | 0.732 |
| Dyspnea | 130 (96.3) | 75 (94.9) | 55 (98.2) | 0.321 |
| DVT symptoms | 27 (20.0) | 17 (21.5) | 10 (17.9) | 0.600 |
| Edema of lower extremities | 27 (20.0) | 17 (21.5) | 10 (17.9) | 0.600 |
| Leg pain | 2 (1.5) | 0 (0.0) | 2 (3.6) | 0.091 |
| Arterial blood gas analysis | | | | |
| PaO_2_/FiO_2_, mm Hg | 137 (80, 188) | 157 (92, 209) | 105 (72, 159) | 0.003 |
| Hematologic and infection-related indices | | | | |
| White blood cell count,  ×10^9^/L | 14.4 (10.1, 19.0) | 14.1 (10.2, 20.4) | 14.8 (10.1, 18.2) | 0.934 |
| Neutrophil count, ×10^9^/L | 12.8 (9.0, 17.3) | 12.8 (8.9, 17.7) | 13.0 (9.1, 16.8) | 0.986 |
| Lymphocyte count, ×10^9^/L | 0.8 (0.5, 1.2) | 0.8 (0.5, 1.3) | 0.8 (0.5, 1.1) | 0.680 |
| Neutrophil-to-lymphocyte ratio | 15.1 (8.7, 23.7) | 15.0 (8.0, 22.9) | 15.3 (11.1, 25.0) | 0.565 |
| Platelet count, ×10^9^/L | 185 (115, 261) | 181 (108, 257) | 192 (127, 266) | 0.765 |
| Hemoglobin, g/L | 112 (87, 130) | 113 (87, 136) | 110 (89, 126) | 0.539 |
| C-reactive protein, mg/L | 120.0 (82.0, 120.0) | 120.0 (85.0, 120.0) | 120.0 (80.5, 120.0) | 0.473 |
| Serum procalcitonin, ng/L | 3.2 (1.2, 11.3) | 2.3 (0.8, 8.6) | 5.2 (1.6, 15.1) | 0.021 |
| Biochemical test | | | | |
| Total protein, g/L | 53.0 (47.0, 59.0) | 53.6 (49.0, 61.0) | 52.4 (46.8, 57.2) | 0.148 |
| Albumin, g/L | 25.3 (23.0, 29.6) | 25.7 (23.5, 30.4) | 24.9 (20.9, 27.6) | 0.072 |
| Aspartate aminotransferase,  U/L | 40.8 (26.0, 71.7) | 44.3 (27.6, 82.6) | 36.7 (25.2, 60.8) | 0.325 |
| Alanine aminotransferase,  U/L | 29.7 (17.9, 58.8) | 33.1 (18.4, 64.9) | 29.1 (15.8, 54.1) | 0.604 |
| Total bilirubin, μmol/L | 15.2 (10.7, 22.8) | 14.8 (10.6, 23.0) | 15.7 (12.0, 22.7) | 0.778 |
| Direct bilirubin, μmol/L | 5.2 (3.3, 8.4) | 5.1 (2.7, 9.2) | 5.3 (3.9, 7.3) | 0.571 |
| Lactate dehydrogenase, U/L | 350.0 (239.0, 624.8) | 339.0 (233.7, 544.0) | 437.4 (255.0, 630.5) | 0.247 |
| Blood urea nitrogen, mmol/L | 10.2 (5.4, 17.4) | 10.2 (5.3, 18.4) | 9.8 (5.7, 15.7) | 0.857 |
| Serum creatinine, μmol/L | 89.1 (62.4, 193.0) | 117.2 (68.0, 221.0) | 78.7 (56.9, 126.8) | 0.015 |
| eGFR, mL/min/1.73m^2^ | 71.6 (29.2, 102.0) | 58.8 (24.0, 102.9) | 75.3 (40.6, 99.9) | 0.195 |
| Creatine kinase isoenzyme  MB, U/L | 16.2 (11.0, 26.9) | 17.2 (11.4, 29.5) | 14.9 (10.4, 24.3) | 0.193 |
| Coagulation function | | | | |
| D-dimer, µg/mL | 1.5 (0.6, 2.6) | 1.2 (0.5, 2.1) | 1.8 (0.8, 3.4) | 0.123 |
| Prothrombin time, s | 13.5 (12.3, 15.2) | 13.5 (12.1, 15.2) | 13.5 (12.4, 15.1) | 0.886 |
| Activated partial  thromboplastin time, s | 32.1 (28.7, 35.7) | 32.2 (29.0, 35.8) | 31.5 (27.3, 35.6) | 0.399 |
| Treatments | | | | |
| Glucocorticoid therapy | 40 (29.6) | 19 (24.1) | 21 (37.5) | 0.092 |
| Immunoglobulin therapy | 3 (2.2) | 2 (2.5) | 1 (1.8) | 0.772 |
| CVC | 45 (33.3) | 26 (32.9) | 19 (33.9) | 0.902 |
| CRRT | 12 (8.9) | 9 (11.4) | 3 (5.4) | 0.225 |
| IMV | 79 (58.5) | 35 (44.3) | 44 (78.6) | < 0.001 |
| Sedative therapy | 62 (45.9) | 28 (35.4) | 34 (60.7) | 0.004 |
| Vasoactive drugs | 27 (20.0) | 15 (19.0) | 12 (21.4) | 0.727 |
| VTE prophylaxis | 64 (47.4) | 41 (51.9) | 23 (41.1) | 0.215 |
| LMWH | 55 (40.7) | 37 (46.8) | 18 (32.1) | 0.087 |
| LMWH + physical | 40 (29.6) | 27 (34.2) | 13 (23.2) | 0.169 |
| Physical prophylaxis only | 8 (5.9) | 4 (5.1) | 4 (7.1) | 0.614 |
| 28-day mortality | 46 (34.)1 | 17 (21.5) | 29 (51.8) | < 0.001 |

Data are presented as mean ± SD, median (IQR), or n (%). *P* values comparing DVT and non-DVT groups were from a two-sample *t-*test, Mann-Whitney *U* test, χ² test, or Fisher exact test. *P* < 0.05 was considered statistically significant.

Abbreviations: APACHE, Acute Physiology and Chronic Health Evaluation; ARDS, acute respiratory distress syndrome; BMI, body mass index; CK, creatine kinase isoenzyme; COVID-19, coronavirus disease 2019; CRRT, continuous renal replacement therapy; CVC, central venous catheterization; DVT, deep venous thrombosis; eGFR, estimated glomerular filtration rate; FiO_2_, a fraction of inspired oxygen; IMV, invasive mechanical ventilation; IQR, interquartile range; LMWH, low molecular weight heparin; PaO_2_, partial pressure of arterial oxygen; SD, standard deviation; SOFA, Sequential Organ Failure Assessment; US, ultrasound; VTE, venous thromboembolism.

**Supplementary Fig. 1** Probability of DVT with serum creatinine levels in the Bacterial pneumonia ARDS group.

There was no association between serum creatinine levels and incidence of DVT in both COVID-19 ARDS group (green line; *P* = 0.221) and bacterial pneumonia ARDS group (green line; *P* = 0.262), the interaction analysis displayed no significant difference between two groups (test for interaction, *P* = 0.363). Data are adjusted for age, serum PCT levels, CK-MB levels, D-dimer levels, PaO_2_/FiO_2_ ratios, and IMV.

Abbreviations: ARDS, acute respiratory distress syndrome; CK, creatine kinase isoenzyme; COVID-19, coronavirus disease 2019; DVT, deep vein thrombosis; FiO_2_, a fraction of inspired oxygen; IMV, invasive mechanical ventilation; PaO_2_, partial pressure of arterial oxygen; PCT, procalcitonin

**Supplementary Fig. 2** Comparison of diagnostic accuracy for screening for DVT of different ROCs in ARDS cohort caused by COVID-19.

We selected the risk factors based on the test results of the Fine-Gray model and proposed combining forecasting models for assessing the risk of DVT in patients with ARDS caused by COVID-19. Patients were split by generating random numbers to produce a training data set (n*0.7) and a validation data set (n*0.3) in ARDS cohort caused by COVID-19. A, the CK-MB level showed satisfactory predicting ability for DVT (AUC = 0.639; 95% CI: 0.428 - 0.850; sensitivity: 70.6%; specificity: 73.3%); yet, there was no significant difference between CK-MB level and the DVT Wells score (AUC = 0.537; *P* = 0.587 for these two curves) and the Padua prediction score (AUC = 0.717; *P* = 0.515 for these two curves) when predicting DVT. B, the CO model, including CK-MB and PaO_2_/FiO_2_ ratio, showed satisfactory predicting ability for DVT (AUC = 0.702; 95% CI: 0.516 - 0.887; sensitivity: 58.8%; specificity: 73.3%); however, there was no significant difference between the CO model and the DVT Wells score (*P* = 0.242 for these two curves) and the Padua prediction score (*P* = 0.888 for these two curves).

Abbreviations: CO = CK-MB + PaO_2_/FiO_2_ ratio; ARDS, acute respiratory distress syndrome; AUC, area under the curve; CI, confidence interval; CK, creatine kinase isoenzyme; DVT, deep vein thrombosis; FiO_2_, a fraction of inspired oxygen; PaO_2_, partial pressure of arterial oxygen; ROC, receiver operating characteristic.

**Supplementary Fig. 3** Nomogram for assessing the risk of DVT.

The nomogram was created based on the selected three predictors. The corresponding points were obtained by making a vertical line upward based on the value of each variable. The total points were obtained by adding the points of the three variables. The probability of DVT in 5-, 7-, and 14 days was obtained by making a vertical line downward based on the total points.

Abbreviations: CK, creatine kinase isoenzyme; DVT, deep vein thrombosis.

**Supplementary Fig. 4** Calibration curve of 5-, 7- and 14 days DVT between the actual observation and the nomogram prediction

The calibration plots showed good consistency of 5-, 7-, and 14 days DVT between the actual observation and the nomogram prediction.

Abbreviations: DVT, deep vein thrombosis.
